# Mentally Sick or Not—(Bio)Markers of Psychiatric Disorders Needed

**DOI:** 10.3390/jcm9082375

**Published:** 2020-07-25

**Authors:** Napoleon Waszkiewicz

**Affiliations:** Department of Psychiatry, Medical University of Białystok, Plac Brodowicza 1, 16-070 Choroszcz, Poland; napoleonwas@yahoo.com

Psychiatric disorders, also called mental illnesses or mental disorders, constitute a wide group of disorders including major depression disorder (MDD), bipolar disorder (BD), schizophrenia (SCZ) and other psychoses, anxiety disorders (ANX), substance-related disorders (SRD), dementia, developmental disorders e.g., autism, etc. They have behavioral and mental patterns, and are characterized by combination of abnormal thoughts, perceptions, emotions, behaviour, and relationships with others. They can be persistent, relapsing/remitting, or can occur as a single episode. Psychiatric disorders significantly worsen the lives of people suffering from them. Even up to one third of the world’s population has some kind of a psychiatric disorder during one year. It was estimated that the prevalence of mental disorders (MDD, ANX, PTSD, BD, and SCZ) was 22.1% at any point in time. The mean comorbidity-adjusted, age-standardised point prevalence was 13.0% for mild forms of MDD, ANX, and PTSD, 4.0% for moderate forms, and 5.1% for severe disorders such as SCZ, BD, severe MDD, severe ANX, and severe PTSD [1]. These disorders affect the entire structure of people’s lives, cause significant distress or impairment of personal functioning, deteriorating quality of life and the course of somatic diseases. The truism is that the diagnosis of mental disorders at an early stage allows for the early inclusion of treatment and/or psychotherapy, before the maximum severity of symptoms and hospitalization occur [1].

Although much has been achieved [2,3,4], generally, no perfect/specific marker of psychiatric disorder has been estabilished so far and medical scientists feel that discovery of such a marker will be one of the most difficult tasks that researchers will ever face. The most important factors that reduce the utility of potential markers can be due to: (a) the fact that levels of some markers can be altered not only by psychiatric but also by neurological disorders, by environmental and lifestyle factors such as stress, diet, level of activity, used psychoactive substances (e.g., alcohol), co-morbidities, or medications (including psychotropic), (b) incorrect/incomparable methodology of research (technical or material-peripheral/central problems), (c) subjective classification of the disorder by clinicians, as the characteristics of psychiatric disorders are multifactorial and multigenic, and we can see a significant overlap of symptoms among these disorders (~30% of bipolar disorders may be initially diagnosed as schizophrenia) [2,3,4].

Despite the abovementioned imperfections of potential psychiatric (bio)markers, they can be used to: (a) identify biologic subtypes of disorders, (b) explain pathophysiologies of disorders, (c) help identify and characterize stages of disorders, (d) predict biological predisposition to disorders, (e) differentiate disorders with overlapping features, (f) identify specific state and trait features, (g) help in developing personalized medicine/treatment including treatment resistance [5].

So far, there have been developed some markers of psychiatric disorders. In MDD, there were found inflammatory (bio)markers such as C-reactive protein (CRP), interleukin-1 (IL-1) and tumor necrosis factor alpha (TNF-α) [3]. However, as Table 1 shows, they are not specific to MDD only. Moreover, patients with three or more failed trials in the current MDD episode had significantly higher plasma TNF, sTNF-R2 and IL-6, so these proteins were promoted as markers of treatment-resistant depression (TRD) [6]. Higher levels of HsCRP can also predict a positive mood response to infliximab. Other potential nine biomarkers of the MDD include α1-antitrypsin, brain-derived-neurotrophic factor (BDNF), apolipoprotein C3, epidermal growth factor (EGF), cortisol, resistin, prolactin, myeloperoxidase, and soluble tumor-necrosis factor α receptor type II (sTNF-αR2) [7]. Some markers, such as BDNF, cytokines, insulin-derived growth factos (IGF), also seem to be applicable to both MDD and TRD diagnosis [2]. To differentiate MDD from BD, some studies suggest to check differences in lactate, alanine, glycine, phenylalanine, tyrosine, sorbitol, pyroglutamate, aminoethanol, and hippurate metabolites when using mass spectroscopy and neuroimaging, as abnormal levels of these potential biomarkers were reported several times. Metabolomic studies also suggest to check the applicability of glutamate, citrate, valine, and formate metabolites in MDD, and eicosapentaenoic acid, 5-hydroxyhexanoic acid, and adipic acid for BD diagnosis/differentiation [8].

Currently, there is an increasing research focus on advances in neuroimaging diagnostics in psychiatry to check if the specific view and metabolite-spectrum can be applicable as markers of psychiatric disorders. However, except for positive [18F] florbetapir-positron emission tomography (PET) signal, that was approved by the U.S. Food and Drug Administration (FDA) for detecting abnormally increased β-amyloid deposition in the brains of patients with cognitive decline, no other specific neuroimaging biomarker of psychiatric disorders has been found so far [9].

A study based on proteomic pointed out six proteins expressed in the brain that could be helpful in differential diagnosis of BD patients from controls [10], whereas some neuroimaging markers can only be helpful in BD (Figure 1) [9].

Patients suffering from SCZ have raised mRNA levels and potential applicability of breath test for ammonia and ethylene [2]. As none of the tests alone is a viable test for SCZ, it was attempted to develop a blood multitest for schizophrenia by using 51 biomarkers (called VeriPsych), but it had an imperfect specificity [11].

Patients suffering from ANX can also be discriminated by using biomarkers at some time point [2]. The startle response during PTSD may be assessed by the cortisol levels [2]. Useful markers in the diagnosis of PTSD may also include low brain natriuretic peptide levels, increased levels of corticotrophin-releasing hormone (CRH) in the cerebrospinal fluid (CSF), or a small right hippocampal volume in neuroimaging [2,12].

As abovementioned, the major dementia biomarkers include neuroimaging tests, which show complete brain atrophy and β-amyloid at PET specific scan [9]. However, in Alzheimer disease (AD), there are also quite useful CSF biomarkers like β-amyloid, tau and phosphotau [13,14].

An increasing number of new biomarkers of alcohol abuse/dependence appear in the literature. The most commonly used biomarkers include 5-hydroxytryptophol, fatty acid ethyl esters (FAEEs), ethyl glucuronide (EtG), phosphatidyl ethanol, ethyl sulphate, aminotransferases (AST/ALT), γ-glutamyl transferase (GGT), carbohydrate-deficient transferrin (CDT), acetaldehyde adducts, beta-hexosaminidase, and sialic acid [15]. Over the last decade there have been considerable developments in the use of oral fluid (saliva) for neuropsychiatric diseases and psychoactive drug testing. Legal and illegal psychoactive substance use/abuse and dependence syndrome can be diagnosed with saliva [16]. Some of them seem to be valuable salivary markers of alcohol use (hexosaminidase A, immune proteins-oral peroxidase and immunoglobulin A), smoking (cotinine-metabolite of nicotine), methadone, cocaine, amphetamines, or buprenorphine use [16,17].

Diagnosis of other disorders such as Attention Deficit Hyperactivity Disorder (ADHD) in children can also be supported by some markers. A new EEG-based diagnostic test (NEBA: Neuro-psychiatric EEG-based Assessment Aid) can be useful in ADHD [18]. In autism, there are found some potential physiological biomarkers that identify neuroimmune and metabolic abnormalities (methylation-redox, acyl-carnitine/amino-acids), neurological biomarkers pointing on abnormalities in brain structure (extra-axial fluid), on function and neurophysiology (EEG N170, mu rhythm, gamma band), on magnetoencephalography (MEG) auditory oscillations, and subtle behavioral biomarkers including atypical development of visual attention, as well as some genetic (single nucleotide polymorphisms) and gastrointestinal biomarkers (zonulin) [19].

As mentioned earlier, morphological changes of the brain can be helpful in differential diagnosis of psychiatric disorders. Figure 1 shows changes in the regional brain structure and activity in different psychiatric disorders [20,21]. It can be assumed that SCZ is characterized by the greatest brain atrophy, MDD by decreased frontal volume, BD by hyperintense frontal signal, OCD by hyperactivity of the orbito-frontal cortex.

Another concept closely-related to markers is an endophenotype. It is a construct based on neurophysiological, biochemical, endocrinological, neuroanatomical, cognitive, or neuropsychological susceptibility to disease, that is repeatable in the family of ill patients, so endophenotype is partly hereditary and determines the susceptibility to falling ill. Endophenotypes may have additional use in psychiatry e.g., in diagnosis, classification, and the development of animal models. To endophenotypes of SCZ we can include sensory motor gating, oculomotor dysfunction, disrupted P300 event-related potential (ERP), or neurocognitive disturbances (working memory/information processing speed, executive function, attention) [22,23].

Although suicide points only on the state, not the disease per se, and can be committed during most psychiatric disorders, many researchers indicate some potentially useful biomarkers for suicidal behaviors such as the peripheral 5HT2A receptor and CSF 5-hydroxyindoleacetic acid (5HIAA), or some of the hypothalamic-pituitary-adrenal (HPA) axis components and cytokines (Table 1) [24].

There are still many fundamental questions that remain unanswered in the aspect of psychiatric (bio)markers, promising a great future for this field. This Special Issue deals with the topic of markers of mental disorders that are undeniably helpful in psychiatric diagnostics. Original research and review articles support us in understanding the importance of markers in psychiatric illness and in personalized medicine. Topics include advances in clinical markers, molecular/biochemical, (neuro)imaging, neurophysiological, neurocognitive markers of psychiatric disorders, as well as advances in markers of successful treatment in psychiatry. Despite methodological difficulties, future research on this subject is essentially needed. The Guest Editor would like to sincerely thank all the authors for their valuable contributions. I hope that the readers enjoy this special issue.

## Figures and Tables

**Figure 1:**
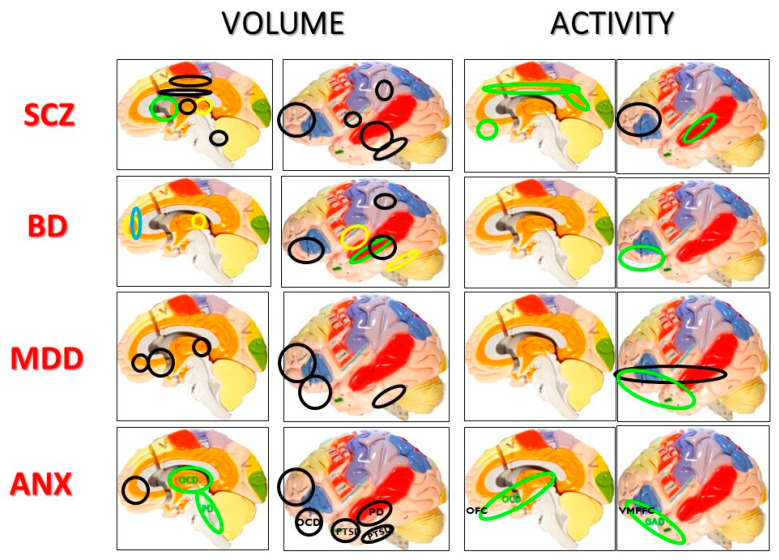
Volume and activity changes of brain areas in psychiatric disorders [20,21] (SCZ—Schizophrenia, BD—bipolar disorder, MDD—Major depression disorder, ANX—Anxiety disorders in general, PTSD—Post-trauma stress disorder, OCD—Obsessive-compulsive disorder, PD—Panic disorder, GAD—Generalized anxiety disorder); OFC—Orbitofrontal cortex, VMPFC—Ventromedial prefrontal cortex; green line—Increase, black line—Decrease, yellow line—Posttreatment increase, blue line—Hyperintense signal.

**Table 1:** Inflammation-related potential blood biomarkers in psychiatric disorders, based on Yuan et al. [3]. ASD—Autism spectrum disorder, BD—Bipolar disorder, CCL—Chemokine Ligand, CRP—C-reactive protein, CXCL—chemokine (CXC motif) ligand, G-CSF—granulocyte colony-stimulating factor, IFN—interferon, IGF-1—insulin-like growth factor 1, IL—interleukin, MDD—Major depression disorder, NGF—nerve growth Figure 100. S100 proteins, SCZ—Schizophrenia, sIL-R—soluble interleukin receptor, SD—Sleeping disorder, TGF—transforming growth factor, TNF—tumor necrosis factor, VEGF—vascular endothelial growth factor; ↑—increase, ↓—decrease, −—no change, ~—inconsistent results, filled-up box with gray—metaanalyzed multiple Times.

	CCL2	CCL3	CCL4	CCL5	CCL11	CRP	CXCL4	CXCL7	G-CSF	IFN-gamma	IGF-1	IL-12p40	IL-1alpha	IL-1beta	IL-1RA	IL-2	IL-4	IL-5	IL-6	IL-8	IL-10	IL-12	IL-13	IL-17	IL-18	IL-23	NGF	NT3	NT4/5	S100	sIL-2R	sIL-6R	sTNF-R1	sTNF-R2	TGF-beta	TGF-beta1	TNF-alpha	VEGF
**MDD**	↑	−	↓		↑	↑	↑	↑		~	↑			−	↑	−	−	−	↑	~	~	↑	↑	−	↑		↓				↑	−		↑	−		~	↑
**BD**	−									−	↑			−	−	−	↑	−	−	−								↑	↑		↑	↑	↑	−			↑	
**SUICIDE**						↑				−						↓	↓		~		↑														↑		−	
**PTSD**						−				↑				↑		−	−		↑	−	−										−	−					−	
**SCZ**						↑				−				−	↑	−	−		↑		−	↑					↓			↑	↑	−					−	
**ASD**	↑		−	−	↑				−	↑		−	−	↑	−		−		↑	↑	−			−		−										↓	−	
**SD**						↑													↑																			
